# Supplementary material for: Plant diversity alters the representation of motifs in food webs
Source: Nat Commun. 2019 Mar 15;10:1226. doi: 10.1038/s41467-019-08856-0 (PMC6420570; doi:10.1038/s41467-019-08856-0)
Supplement: Supplementary file 1 — Supplementary Information [file 41467_2019_8856_MOESM1_ESM.pdf]

nature research

Corresponding author(s): Darren Giling
Last updated by author(s): Jan 18, 2019

Reporting Summary

Nature Research wishes to improve the reproducibility of the work that we publish. This form provides structure for consistency and transparency in reporting. For further information on Nature Research policies, see Authors & Referees and the Editorial Policy Checklist.

Statistics

For all statistical analyses, confirm that the following items are present in the figure legend, table legend, main text, or Methods section.

n/a	Confirmed	
☐	☒	The exact sample size (*n*) for each experimental group/condition, given as a discrete number and unit of measurement
☐	☒	A statement on whether measurements were taken from distinct samples or whether the same sample was measured repeatedly
☐	☒	The statistical test(s) used AND whether they are one- or two-sided<br>*Only common tests should be described solely by name; describe more complex techniques in the Methods section.*
☐	☒	A description of all covariates tested
☐	☒	A description of any assumptions or corrections, such as tests of normality and adjustment for multiple comparisons
☐	☒	A full description of the statistical parameters including central tendency (e.g. means) or other basic estimates (e.g. regression coefficient) AND variation (e.g. standard deviation) or associated estimates of uncertainty (e.g. confidence intervals)
☐	☒	For null hypothesis testing, the test statistic (e.g. *F*, *t*, *r*) with confidence intervals, effect sizes, degrees of freedom and *P* value noted<br>*Give P values as exact values whenever suitable.*
☒	☐	For Bayesian analysis, information on the choice of priors and Markov chain Monte Carlo settings
☐	☒	For hierarchical and complex designs, identification of the appropriate level for tests and full reporting of outcomes
☒	☐	Estimates of effect sizes (e.g. Cohen's *d*, Pearson's *r*), indicating how they were calculated

Our web collection on statistics for biologists contains articles on many of the points above.

Software and code

Policy information about availability of computer code

Data collection	Custom code used to generate the data is available on GitHub with a DOI minted by figshare (https://doi.org/10.6084/m9.figshare.7605173.v2).
Data analysis	All analyses were conducted in the statistical software R using the packages described in the Methods text. Custom code used is available on GitHub (https://doi.org/10.6084/m9.figshare.7605173.v2).

For manuscripts utilizing custom algorithms or software that are central to the research but not yet described in published literature, software must be made available to editors/reviewers. We strongly encourage code deposition in a community repository (e.g. GitHub). See the Nature Research guidelines for submitting code & software for further information.

Data

Policy information about availability of data

All manuscripts must include a data availability statement. This statement should provide the following information, where applicable:

- Accession codes, unique identifiers, or web links for publicly available datasets
- A list of figures that have associated raw data
- A description of any restrictions on data availability

The datasets generated and analysed during the current study (including raw data for Figs. 2 & 3 and Supplementary Figs.) are available in the figshare repository (https://doi.org/10.6084/m9.figshare.7605242.v2). The metaweb that supports the findings of this study is available in reference 21.

Field-specific reporting

Please select the one below that is the best fit for your research. If you are not sure, read the appropriate sections before making your selection.

☐ Life sciences ☐ Behavioural & social sciences ☒ Ecological, evolutionary & environmental sciences

For a reference copy of the document with all sections, see nature.com/documents/nr-reporting-summary-flat.pdf

Ecological, evolutionary & environmental sciences study design

All studies must disclose on these points even when the disclosure is negative.

Study description	This study tests the effect of plant diversity on the prevalence of trophic motifs in aboveground food webs. Data were collected from a long-term biodiversity experiment (The Jena Experiment). The design is nested (total of 80 plots nested within four experimental blocks) and measurements were repeated over time (four time periods, total n = 320). Blocks are arranged corresponding to variation in soil conditions perpendicular to the nearby Saale River.
Research sample	Each data point is a characteristic of a food web identified on a single plot at one point in time. These food webs represent the structure of feeding interactions occurring among grassland plants and arthropods (from classes Arachnida, Insecta, Diplopoda and Chilopoda) that were identified on that plot. The plant communities were manipulated and maintained while arthropod species could colonise openly from the surrounds.
Sampling strategy	Plants were identified by visual inspection and arthropods were collected with pitfall traps (for ground-dwelling taxa) and suction sampling (for herb-layer species). No sample size calculation was performed, and sample sizes were chosen based on the level of variation observed during similar sampling campaigns in earlier years of the experiment.
Data collection	Data pertaining to the plant and arthropod communities was collected in the field and lab by a subset of the authors (AE, NE, STM, CR, MR, WV, WW). Identification of arthropod species was performed by microscopic inspection by specialist taxonomists, as listed in Hines et al. (in press)
Timing and spatial scale	Samples were collected twice per year (2010 and 2012) during May/June and July, which represents the main growing season and covers the period during which different arthropod species have active aboveground life stages. Precise dates of all sampling periods are available in Supplemental Table 1 of Hines et al. (in press), which is provided as a relevant manuscript file.
Data exclusions	Arthropod species for which less than three individuals were captured across the entire experiment in a given year were excluded from analyses. This was because these extremely rare species contribute little to the structure of the consumer communities and obtaining accurate trait information is highly challenging.
Reproducibility	It was not possible to reproduce this study because it is a large-scale and long-term experiment. Results were consistent among years.
Randomization	Suction samples were placed randomly within the experimental plots.
Blinding	Blinding was not possible during collection of samples from the experimental field site. Plot codes used during data input and supplied to taxonomists do not identify the level of plant species richness.

Did the study involve field work? ☒ Yes ☐ No

Field work, collection and transport

Field conditions	Fieldwork was conducted between 9am and 5pm during May, June and July in dry conditions
Location	The field site (The Jena Experiment) is located near Jena, Germany (50°55`N, 11°35`E, 130 m a.s.l.)
Access and import/export	Plant and arthropod sampling was conducted with the permission of the city council of Jena, Germany.
Disturbance	No natural ecosystems were disturbed in this study. The experiment was established on a previously arable field in 2002, creating no additional disturbance due to change in land-use.

Reporting for specific materials, systems and methods

We require information from authors about some types of materials, experimental systems and methods used in many studies. Here, indicate whether each material, system or method listed is relevant to your study. If you are not sure if a list item applies to your research, read the appropriate section before selecting a response.

Materials & experimental systems

n/a	Involved in the study
☒	☐ Antibodies
☒	☐ Eukaryotic cell lines
☒	☐ Palaeontology
☐	☒ Animals and other organisms
☒	☐ Human research participants
☒	☐ Clinical data

Methods

n/a	Involved in the study
☒	☐ ChIP-seq
☒	☐ Flow cytometry
☒	☐ MRI-based neuroimaging

Animals and other organisms

Policy information about studies involving animals; ARRIVE guidelines recommended for reporting animal research

Laboratory animals	Did not involve laboratory animals
Wild animals	A total of 403 grassland arthropod species were collected from the experiment. These were caught in pitfall traps (euthanized by 3% formaldehyde solution in trap and subsequently stored in 70% ethanol solution) and by suction sampling (euthanized and preserved in 70% ethanol solution). Preservation was necessary for identification. A full species list is available in Hines et al. (in press).
Field-collected samples	Did not involve use of live field-collected organisms.
Ethics oversight	No ethical approval was required to work with invertebrates

Note that full information on the approval of the study protocol must also be provided in the manuscript.
