# Supplementary material for: Plant diversity alters the representation of motifs in food webs
Source: Nat Commun. 2019 Mar 15;10:1226. doi: 10.1038/s41467-019-08856-0 (PMC6420570; doi:10.1038/s41467-019-08856-0)
Supplement: Supplementary file 3 — Reporting Summary [file 41467_2019_8856_MOESM3_ESM.pdf]

Reviewers' Comments:

Reviewer #2:
Remarks to the Author:
I was referee #2 for a previous version of this manuscript. I feel the authors have done an excellent job at addressing my previous comments. They have now included a well-balanced discussion of the relationship between motif representation and potential impacts on food-web stability. They also make clear the limitations of their type of data in making inferences about species interactions. I appreciate the extra effort the authors put in to showing that their inferences are robust to different underlying assumptions when generating these networks. I also found their analysis of motifs in consumer sub webs to be an exciting addition, as it highlights important indirect effects of plant species diversity. Taken together, this work represents an original integration of biodiversity-ecosystem function (BEF) and food-web research that I expect will be of interest to a broad audience.

Reviewer #4:
Remarks to the Author:
The authors have made significant changes since the last version, and the manuscript has improved substantially. In particular, the selection of an adequate model to randomize the network and to lower the claims about the consequences for food web stability. This is now a more robust and honest paper.

**NCOMMS-18-32800-T**

**RESPONSE TO REVIEWERS' COMMENTS:**

Reviewer #2 (Remarks to the Author):

I was referee #2 for a previous version of this manuscript. I feel the authors have done an excellent job at addressing my previous comments. They have now included a well-balanced discussion of the relationship between motif representation and potential impacts on food-web stability. They also make clear the limitations of their type of data in making inferences about species interactions. I appreciate the extra effort the authors put in to showing that their inferences are robust to different underlying assumptions when generating these networks. I also found their analysis of motifs in consumer sub webs to be an exciting addition, as it highlights important indirect effects of plant species diversity. Taken together, this work represents an original integration of biodiversity-ecosystem function (BEF) and food-web research that I expect will be of interest to a broad audience.
**RESPONSE: Thank you for the constructive feedback that enabled us to improve the clarity and robustness of our analysis.**

Reviewer #4 (Remarks to the Author):

The authors have made significant changes since the last version, and the manuscript has improved substantially. In particular, the selection of an adequate model to randomize the network and to lower the claims about the consequences for food web stability. This is now a more robust and honest paper.
**RESPONSE: Thank you, we appreciate the reviewer’s helpful comments on the previous version of this manuscript.**
